# Spread and Impact of COVID-19 in China: A Systematic Review and Synthesis of Predictions From Transmission-Dynamic Models

**DOI:** 10.3389/fmed.2020.00321

**Published:** 2020-06-18

**Authors:** Yi-Fan Lin, Qibin Duan, Yiguo Zhou, Tanwei Yuan, Peiyang Li, Thomas Fitzpatrick, Leiwen Fu, Anping Feng, Ganfeng Luo, Yuewei Zhan, Bowen Liang, Song Fan, Yong Lu, Bingyi Wang, Zhenyu Wang, Heping Zhao, Yanxiao Gao, Meijuan Li, Dahui Chen, Xiaoting Chen, Yunlong Ao, Linghua Li, Weiping Cai, Xiangjun Du, Yuelong Shu, Huachun Zou

**Affiliations:** ^1^School of Public Health (Shenzhen), Sun Yat-sen University, Shenzhen, China; ^2^School of Mathematical Sciences, Queensland University of Technology, Brisbane, QLD, Australia; ^3^Kirby Institute, University of New South Wales, Sydney, NSW, Australia; ^4^School of Public Health, Sun Yat-sen University, Guangzhou, China; ^5^Department of Internal Medicine, University of Washington, Seattle, WA, United States; ^6^State Key Laboratory of Food Nutrition and Safety, Tianjin University of Science and Technology, Tianjin, China; ^7^College of Food Science and Technology, Tianjin University of Science and Technology, Tianjin, China; ^8^Guangzhou Eighth People's Hospital, Guangzhou Medical University, Guangzhou, China; ^9^Shenzhen Center for Disease Control and Prevention, Shenzhen, China; ^10^School of Public Health, Shanghai Jiao Tong University, Shanghai, China

**Keywords:** the reproduction number, incubation, infectious period, fatality, mathematical model

## Abstract

**Background:** Coronavirus disease 2019 (COVID-19) was first identified in Wuhan, China, in December 2019 and quickly spread throughout China and the rest of the world. Many mathematical models have been developed to understand and predict the infectiousness of COVID-19. We aim to summarize these models to inform efforts to manage the current outbreak.

**Methods:** We searched PubMed, Web of science, EMBASE, bioRxiv, medRxiv, arXiv, Preprints, and National Knowledge Infrastructure (Chinese database) for relevant studies published between 1 December 2019 and 21 February 2020. References were screened for additional publications. Crucial indicators were extracted and analysed. We also built a mathematical model for the evolution of the epidemic in Wuhan that synthesised extracted indicators.

**Results:** Fifty-two articles involving 75 mathematical or statistical models were included in our systematic review. The overall median basic reproduction number (R_0_) was 3.77 [interquartile range (IQR) 2.78–5.13], which dropped to a controlled reproduction number (R_c_) of 1.88 (IQR 1.41–2.24) after city lockdown. The median incubation and infectious periods were 5.90 (IQR 4.78–6.25) and 9.94 (IQR 3.93–13.50) days, respectively. The median case-fatality rate (CFR) was 2.9% (IQR 2.3–5.4%). Our mathematical model showed that, in Wuhan, the peak time of infection is likely to be March 2020 with a median size of 98,333 infected cases (range 55,225–188,284). The earliest elimination of ongoing transmission is likely to be achieved around 7 May 2020.

**Conclusions:** Our analysis found a sustained R_c_ and prolonged incubation/ infectious periods, suggesting COVID-19 is highly infectious. Although interventions in China have been effective in controlling secondary transmission, sustained global efforts are needed to contain an emerging pandemic. Alternative interventions can be explored using modelling studies to better inform policymaking as the outbreak continues.

## Introduction

An outbreak of atypical pneumonia (Coronavirus Disease 2019, COVID-19) caused by the novel coronavirus (2019-nCoV) emerged in Wuhan, China, at the end of 2019. The virus rapidly spread across China and the rest of the world. As of 9 May, 83,976 confirmed infections and 4,639 deaths had been reported within China^1^^,^^2^. The majority of cases in China have been identified in Hubei Province, especially within Wuhan. The Wuhan Municipal Government began a citywide lockdown on 23 January 2020 to slow the spread of the disease, and other cities in Hubei Province soon followed suit^3^. The lockdown effectively curbed further exportation of the epidemic from Hubei to the other provinces of China (1–4). Within China, the outbreak has been effectively under control and the main effort was put in identifying the imported cases from overseas^1^. However, the WHO declared COVID-19 a global pandemic on 11 March due to its spread and severity worldwide, with 2,361,998 confirmed infections and 272,094 deaths outsides China as of 9 May.

Mathematical modelling, including statistical modelling, is a useful tool to understand the dynamics of new diseases. Since COVID-19 was first identified, many mathematical models have been developed to simulate the transmission across populations and assess the potential impact of public health interventions. Crucial parameters of new diseases can be derived from models, including the basic reproduction number (R_0_), peak time, peak size, incubation period, infectious period, case-fatality rate (CFR), and elimination time. By definition, R_0_ measures the average number of secondary cases that are expected to be generated from a single case of a disease entering a completely susceptible population (5). R_0_ decreases if intervention measures are implemented or the susceptible population size decreases. The controlled reproduction number (R_c_) denotes R_0_ after interventional measures are undertaken. If R_0_ < 1, then one infectious person will infect fewer than one person, and an epidemic will ultimately resolve (6). Thus, R_0_ is an important parameter to assess potential control strategies during an outbreak. Peak time refers to the time when a disease infects the largest number of people (peak size) and is an inflection point during an outbreak.

Published models of COVID-19 have reported a huge range of estimated R_0_ and peak times. For example, Zhang et al. (7) estimated an R_0_ of 1.44 while Mizumoto et al. (8) reported an R_0_ of 7.05. To better inform efforts to control the current outbreak, we systematically reviewed existing mathematical and statistical models and built our own mathematical model to estimate the transmission capacity, epidemiological characteristics, potential peak time and size, and elimination time of COVID-19.

## Methods

### Search Strategy and Selection Criteria

Our systematic review was conducted according to PRISMA guidelines (9). We searched PubMed, Web of Science, EMBASE, bioRxiv, medRxiv, arXiv, Preprints, and National Knowledge Infrastructure (CNKI) for studies published between 1 December 2019 and 21 February 2020. We used the search terms “Coronavirus Disease 2019,” “COVID-19,” “SARS-CoV-2,” “2019-nCoV,” “coronavirus,” OR “pneumonia” AND “model,” “modelling,” “modeling,” “dynamic,” “estimation,” “prediction,” OR “transmission.” Search terms were translated into Chinese when searching Chinese database. The database search was supplemented by screening references of retrieved articles.

Studies were included if they presented a mathematical/statistical model of COVID-19 and reported any of the following—R_0_, incubation period, infectious period, fatality, peak time, peak size, total infection number, or elimination time. Studies were excluded if they were purely methodological and did not report the aforementioned parameters. If one study was concurrently published in a journal and preprint website, only the journal version was included. Two reviewers (YL and YZ) independently performed the literature search and screened titles and abstracts. Disagreements were resolved by discussion among all authors.

### Data Analysis

Data extraction was performed by QD, Y-FL, and YZho independently, and results were summarised by Y-FL. Abstracted variables included the first author, model type, type and period of data used for model fitting, setting, region of interest, estimated R_0_, estimated incubation period, estimated CFR, estimated peak time and peak size, and impact of outbreak response if available. Assumed values of the incubation period based on other studies were excluded. Quality of mathematical models was assessed according to a quality-appraisal tool developed upon the recommendations by the International Society for Pharmacoeconomics and Outcomes Research and Society for Medical Decision Making (ISPOR-SMDM) Modelling Good Research Practices Task Force (10, 11). Such a tool brings up questions regarding 14 criteria, e.g., model setting and population, modelling methodology and structure, and fitting methodology (see Appendix in Supplementary Material). Each criterion of a paper was scored zero, one, or two. If a criterion was not relevant for a paper, then a score of one was assigned. QD, Y-FL, and YZho assessed the quality of mathematical models, and discrepancies were resolved through discussion with a senior investigator (HZo).

A comprehensive meta-analysis of extracted data was not performed due to the high level of heterogeneity between the studies in terms of model type, model setting, type and period of data used for model calibration, and region of interest. However, some key parameters were analysed, including estimated parameters (R_0_/R_c_, incubation period, infectious period, and CFR) and model predictions (peak size and peak time, total infections, and elimination time). R_0_/R_c_ was analysed by stratifying regions, namely “Wuhan,” “Hubei (including Wuhan),” “mainland China (excluding Taiwan, Hong Kong and Macau),” and “regions other than Hubei in mainland China.”

We reported distributions of point estimates and reported medians and interquartile ranges (IQR). We did not pool point estimates from various mathematical models. Categorical variables were presented as frequencies or proportions. Data were combined by interval segments and/or grouped by the same characteristics according to the sparsity of data. Some statistical methods, mainly non-parametric methods, including the Wilcoxon rank sum test (R_0_/R_c_), Kruskal-Wallis H test (regions), and Quade test (R_0_/R_c_ and regions), were used to analyze differences between segments/groups.

We modelled COVID-19 transmission using a classic susceptible (S)-exposed (E)-infectious (I)-recovery (R) (SEIR) structure model (see Appendix in Supplementary Material) to predict future trends and expected peak time in Wuhan. Two assumptions were separately considered in this SEIR model: (1) individuals in the incubation period are infectious and (2) individuals in the incubation period are not infectious. We used the parameter values that were obtained from our review of previous models for our model simulations. When calibrating the model, the top 20 of 256 best-fit simulations, selected by least square error, were used to obtain estimates of epidemic trends. We calculated peak times and eliminations (total infections <100) based on normal (median), optimistic, and pessimistic scenarios. All analyses were conducted with R software version 3.6.2 (R Foundation for Statistical Computing). Confirmed reported cases between 12 February 2020 and 21 February 2020 were downloaded from the China National Health Commission website ^1^.

## Results

**Figure 1:**
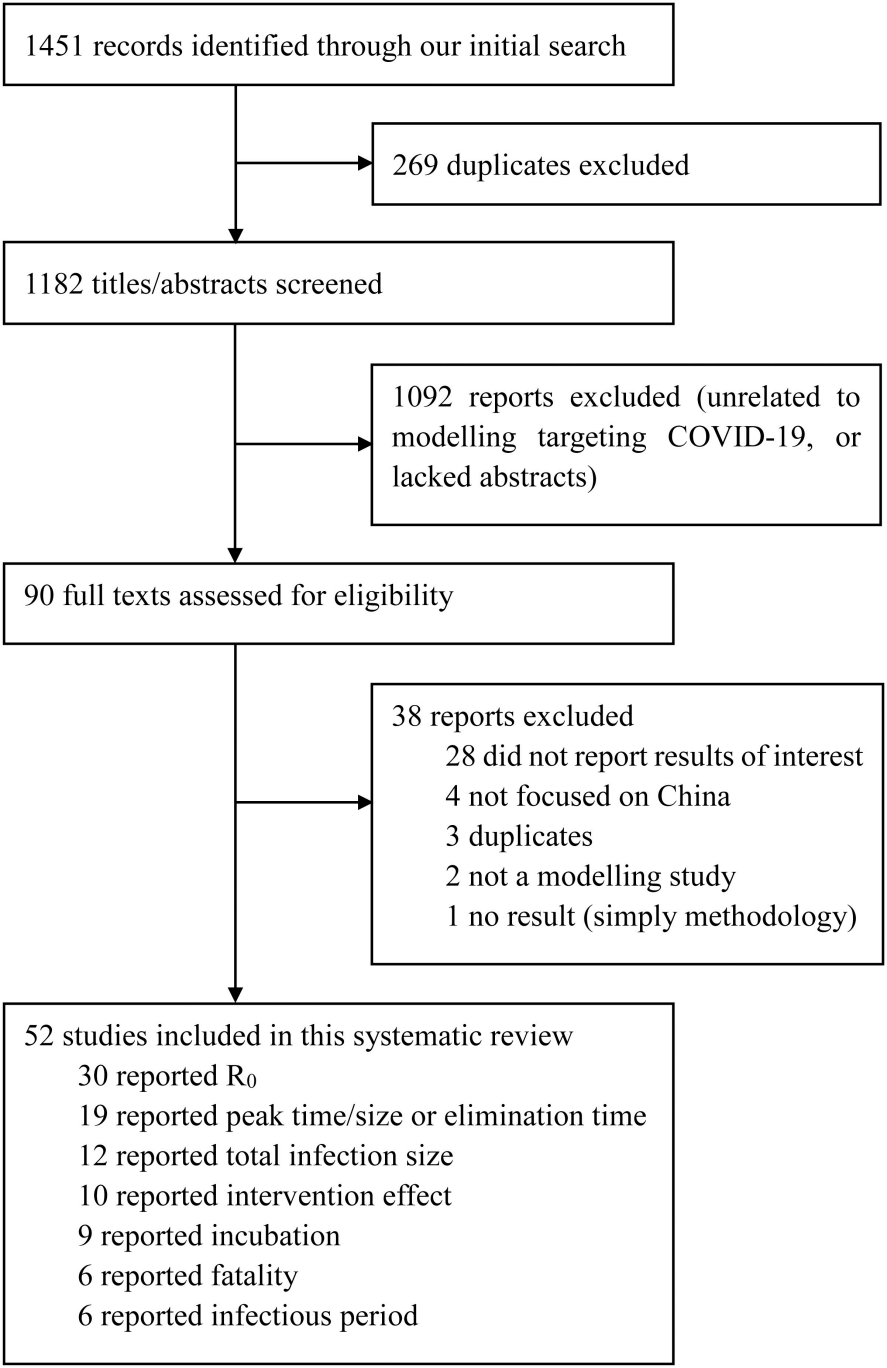
Selection of reports for inclusion in systematic review. Coronavirus Disease 2019, COVID-19; R0, the basic reproduction number.

We identified 1,451 studies; 269 were duplicates, which left us with 1,182 unique studies (Figure 1). After screening titles and abstracts, 90 studies underwent a full-text review. Of these, 38 were excluded because they did not report necessary parameters or were not models specifically targeting COVID-19. Fifty-two publications were eligible for inclusion. Details of each included study is summarised in the Appendix (Supplementary Material) (4, 7, 8, 12–36).

The 52 included studies reported a total of 75 unique models, including 88% (66/75) of articles calibrated models using original data (i.e., reported cases), 7% (5/75) used adjusted data modified by reported rates prior to model fitting, and 5% (4/75) articles used simulations. 35% (26/75), 16% (12/75), 37% (28/75), and 12% (9/75) of models refer to Wuhan, Hubei, mainland China, and regions other than Hubei in mainland China. The other regions mentioned in these nine articles were too wide, such as Guangdong Province, Beijing, and Chongqing, so we decided to focus on the first three regions only. The usage frequency of different models is summarised in the Appendix (Supplementary Material).

**Figure 2:**
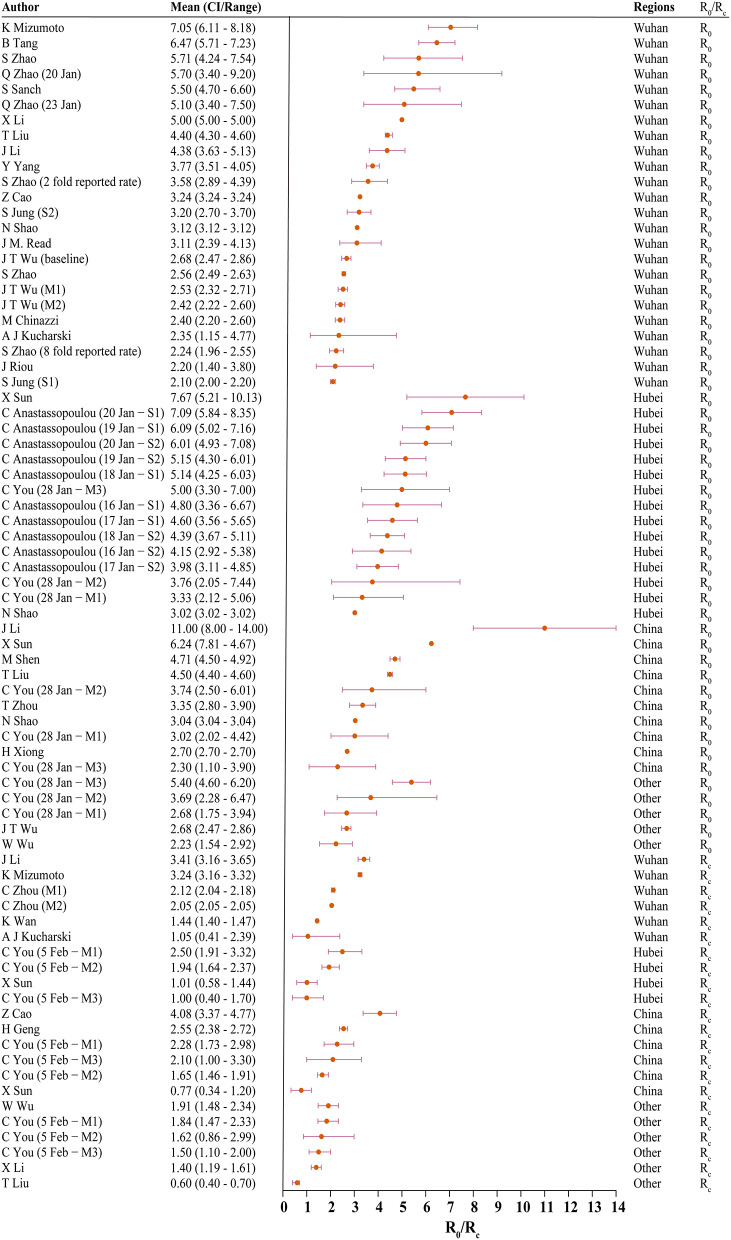
The basic reproduction number (R0) and controlled reproduction number (Rc) estimated among models. CI, confidence interval; M1, model 1; M2, model 2; S1, scenario 1; S2, scenario 2; Other, regions other than Hubei in China.

**Figure 3:**
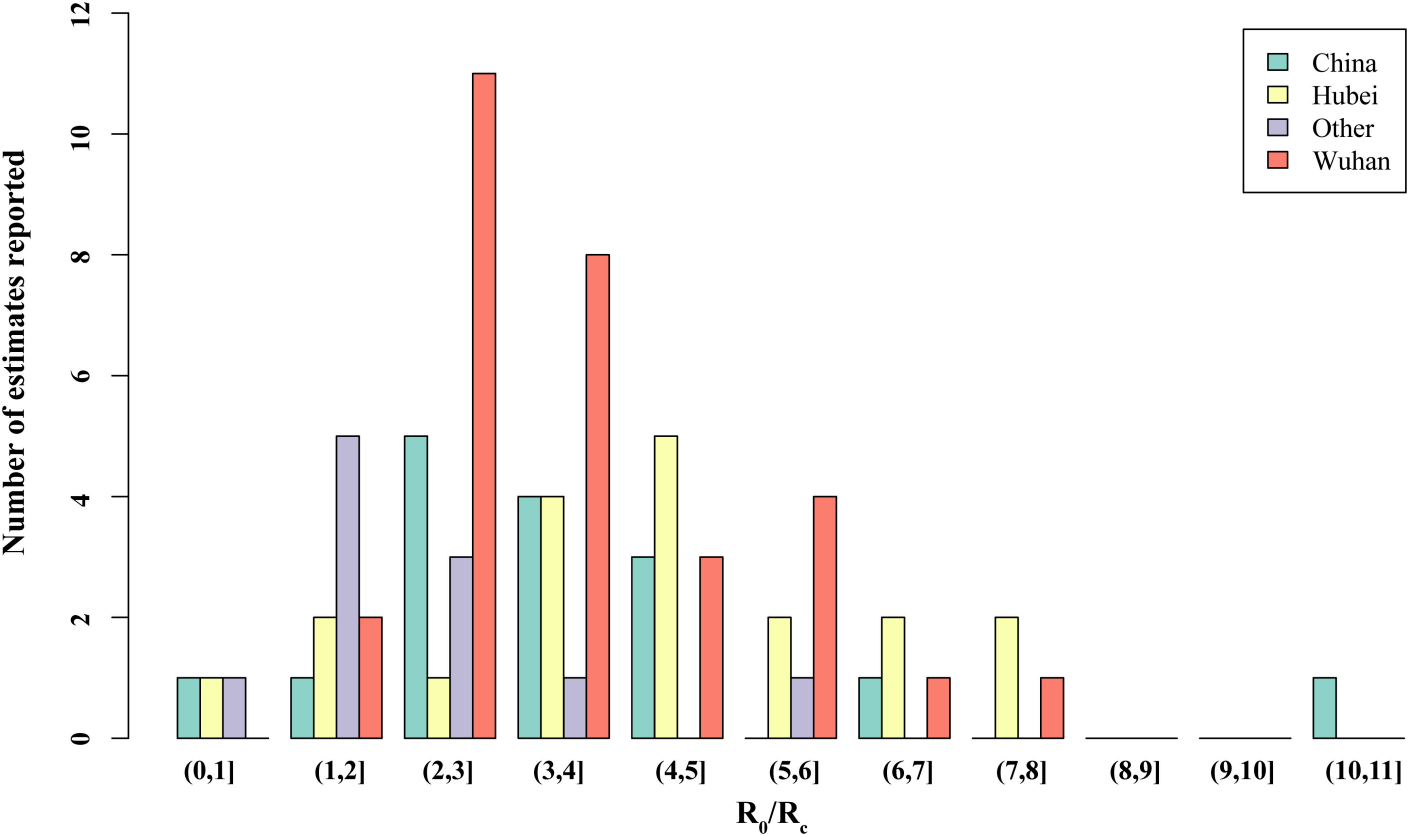
Distribution of the basic reproduction number (R0) and controlled reproduction number (Rc) estimated among models. Other, regions other than Hubei in China.

Thirty studies reported estimated R_0_ and/or R_c_. Models used to generate R_0_/R_c_ varied in terms of model type, model structure, model setting, and data used for model fitting (see Appendix in Supplementary Material). R_0_ differed significantly before and after the citywide lockdown in Wuhan (Figures 2, 3, *p* < 0.001). The median R_0_ was 3.77 (IQR, 2.78–5.13), and median R_c_ was 1.88 (IQR, 1.41–2.24). After aggregating data by regions, median R_0_ for Wuhan, Hubei, and mainland China over the whole outbreak period were 3.16 (IQR, 2.36–4.40), 4.39 (IQR, 3.18–5.15), and 3.03 (IQR, 2.30–4.19), respectively. Differences between these estimated R_0_ were not statistically significant (*p* = 0.180).

When R_0_ and R_c_ were stratified by region, differences in R_0_ and R_c_ remained statistically significant across all four regions. In Wuhan, the median of R_0_ was 3.22 (IQR, 2.50–5.03) and R_c_ was 2.09 (IQR, 1.95–2.96). In Hubei, the median R_0_ and R_c_ were 4.80 (IQR, 4.07–5.58) and 1.48 (IQR, 1.01–2.08), respectively. Across mainland China, R_0_ was 3.55 (IQR, 3.03–4.66), and the median R_c_ was 2.19 (IQR, 1.76–2.48).

Nineteen studies predicted peak time and peak size of infections. Estimates varied from late January to late March with peak size ranging from 7,000 to 90,000 (Table 1). Elimination time was predicted in five studies, with estimates ranging from March to August 2020 (7, 37–40).

**Table 1:** Peak time/size and elimination time predicted in models.

**First author**	**Model**	**Peak time**	**Peak size**	**Elimination**	**Location**
Zhu	ODE based: SIR model	Still goes up/10 February/middle or late with work/school resuming	NA	NA	Other^*^
Wang	ODE based: SIR model	10 March	NA	NA	China
Wu	ODE based: SIR model	17 March	NA	NA	Other
Xiong	ODE based: EIR model (100% Quarantined rate)	16 February	49,093	NA	China
Xiong	ODE based: EIR model (90% Quarantined rate)	17 February	51,605	NA	China
Xiong	ODE based: EIR model (80% Quarantined rate)	18 February	55,059	NA	China
Xiong	ODE based: EIR model (70% Quarantined rate)	19 February	59,953	NA	China
Xiong	ODE based: EIR model (63% Quarantined rate)	20 February	64,740	NA	China
Tang	ODE based: SEIR model	10 February	163,000	NA	China
Wang	ODE based: SEIR model (R_0_ = 0.5)	5 February	11,966	NA	China
Wang	ODE based: SEIR model (R_0_ = 0.25)	4 February	11,373	NA	China
Wang	ODE based: SEIR model (R_0_ = 0.125)	3 February	11,116	Early May	China
Wu	ODE based: SEIR model	April	NA	NA	Wuhan
Wu	ODE based: SEIR model	Mid-February	NA	NA	China
Ai	ODE based: SEIR model	28 January−7 February	7,000–9,000	NA	Hubei
Peng	ODE based: SEIR model	NA	NA	Beginning April	Wuhan
Peng	ODE based: SEIR model	NA	NA	Mid-March	Hubei
Wan	ODE based: SEIR model	19 February	45,000	Late March	Wuhan
Wan	ODE based: SEIR model	9 March (2–24 March)	313,00 (27,700–36,800)	NA	China (without Hubei)
Wan	ODE based: SEIR model	3 March (27 February−18 March)	63,800 (59,300–76,500)	NA	Hubei
Li	ODE based: SEIR model	10 March (19 February−30 March)	NA	NA	Wuhan
Li	ODE based: SEIR model	31 March (15 March−16 April)	NA	NA	Other
Liu	ODE based: Flow-SEIR model	9 March (2–24 March)	85,500 (76,700–97,500)	1.5–2 months from the peak	China
Liu	ODE based: Flow-SEIR model	29 February (25 February−8 March)	62,800 (56,900–70,300)	1.5–2 months from the peak	Hubei
Shen	ODE based: SEIJR model (isolation)	Early-March (1 March)	827 (421–1232)	NA	China
Shen	ODE based: SEIJR model (lockdown)	17 February (14–27 February)	12,143 (5,872–19,852)	NA	China
Zeng	ODE based model	NA	NA	28 February	China
Zeng	ODE based model	NA	NA	10 March	China
Zeng	ODE based model	NA	NA	29 February	China
Zeng	ODE based model	NA	NA	24 February	China
Zeng	ODE based model (NN-−1day delay)	NA	NA	28 February	China
Zeng	ODE based model (NN-−2 days delay)	NA	NA	3 March	China
Zeng	ODE based model (NN—no policies)	NA	NA	28 April	China
Batista	Probabilistic/likelihood-based model	4 February	NA	NA	China
Batista	Probabilistic/likelihood-based model	22 August	NA	NA	China
Hermanowicz	EG model	7–20 February	65,000	NA	China
Liu	EG model	4 February	NA	NA	Wuhan

* *Other regions other than Hubei in China*.

*ODE, Ordinal Differential Equation; SIR, Susceptible-Infected-Recovered; EIR, Exposed-Infectious-Recovered; SEIR, Susceptible-Exposed-Infectious-Recovered; SEIJR, Susceptible-Exposed-Infectious-Isolated-Recovered; EG, Exponential Growth; R_0_, the reproduction number*.

**Figure 4:**
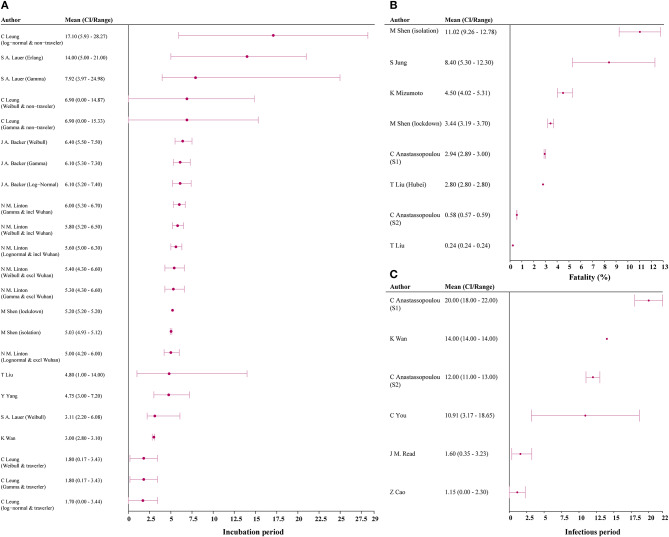
Incubation, case-fatality rate, and infectious period estimates among models. **(A)** incubation period; **(B)** fatality; **(C)** infectious period. CI, confidence interval; S1, scenario 1; S2, scenario 2.

Incubation period was estimated in 9 studies, with the median estimate being 5.90 days (IQR 4.78–6.25) (3, 7, 20, 22, 28, 41–44). Among the six studies reporting infection period, the median estimate was 9.94 days (IQR, 3.93–13.50) [Figure 4; (7, 14, 15, 17, 25)]. Six studies reported CFR, and median estimated CFR was 2.94% (IQR, 2.25%−5.40%) (3, 8, 15, 20, 22, 31).

The estimated number of total infections varied by region (see Appendix in Supplementary Material). Median estimated number of total infections in Wuhan, Hubei, and mainland China were 56,565 (IQR, 49,795–280,255), 61,028 (IQR, 43,750–111,682), and 87,525 (IQR, 59,784–461,652), respectively (3, 8, 15, 17, 20, 38, 39, 45–48). Most studies used data from 23 January 2020 or earlier when building models. Two thirds of studies that predicted abnormally high totals came from Probabilistic/likelihood-based models that used data from after 23 January 2020 (15, 46, 48).

The estimated impact of interventions is summarised in the Appendix (Supplementary Material). Four of 10 studies found that after implementing citywide lockdown in Wuhan, R_0_ would be reduced by 87–95%, peak size would be reduced by 21.06–22.38%, and deaths would be reduced by 56.87–62.95% (1–4). Three studies predicted delay of lockdown measures by 1 or 7 days would increase the number of infections at peak size by 722–6,351 and 8,618–28,274, respectively (3, 16, 49). Increasing diagnosis efficacy to 70% was predicted to reduce infections by 90% as of 10 February 2020 (50).

We generated models under two separate assumptions: (A1) individuals are infectious during the incubation period and (A2) individuals are not infectious during the incubation period. In the A1 model, mean R_c_ was 2.15 (SD, 0.15); mean incubation period was 5.19 days (SD, 0.53); mean infectious period was 11.87 days (SD, 1.35); and CFR was 2.68% (SD, 0.67%). In the A2 model, mean R_c_ was 2.14 (SD, 0.16); mean incubation period was 5.17 days (SD, 0.50); mean infectious period was 12.00 days (SD, 1.51); and CFR was 2.43% (SD, 0.39%). Mean of least square error in the top 20 best-fit simulations was 17,944 (SD, 1,140) and 27,750 (SD, 1,754) in the A1 and A2 models, respectively (see Appendix in Supplementary Material).

**Figure 5:**
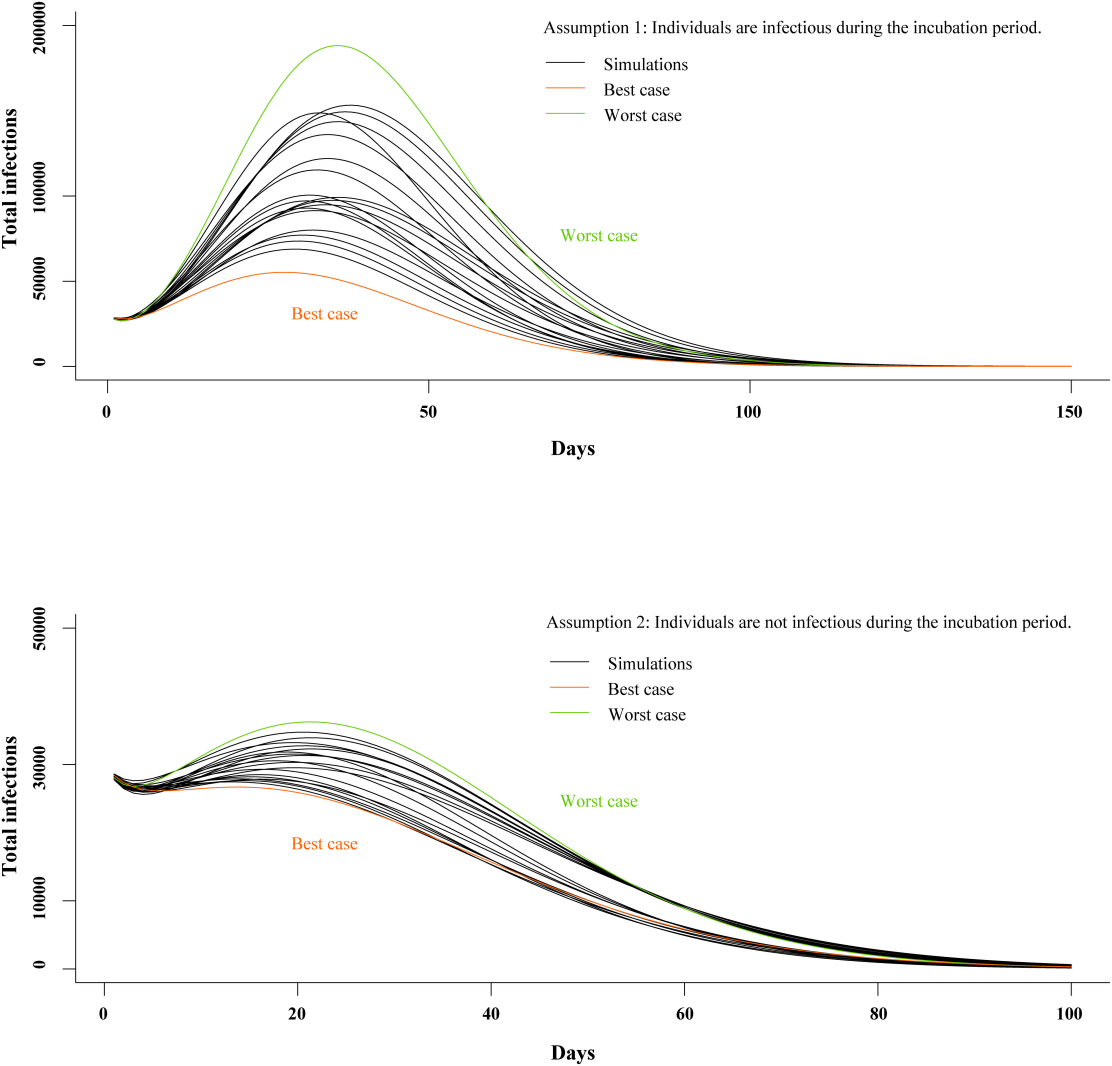
Estimates of peak time and elimination time in SEIR model. SEIR, susceptible-exposed-infectious-recovered.

The top 20 best-fit simulations with best- and worst-case scenarios are presented in Figure 5. In the A1 model, R_c_, peak size was 55,225 and 188,284 in the best- and worst-case scenarios, respectively. In the A2 model, peak size was 28,237 and 36,248 in the best- and worst-case scenarios, respectively. In Wuhan, the A1 model predicted peak time would be 17 March (Range, 12–22 March 2020), and elimination time would be 7 May (25 April−21 May 2020). In the A2 model, peak time was estimated to be 2 March (Range, 13 February−5 March 2020) and elimination time to be 17 May (8–27 May 2020).

## Discussion

Our systematic review and data synthesis is the first study to synthesize mathematical models on the transmission of COVID-19. The estimated values of R_0_, incubation period, infectious period, peak time, and peak size for COVID-19 were consistently higher than that of SARS or MERS, suggesting this novel coronavirus is highly infectious. We also found that the citywide lockdown of Wuhan resulted in significantly reduced R_0_, with the earliest elimination time in China now estimated to be late April, though the complex dynamics of an evolving global pandemic were not incorporated into included models.

In this systematic review of transmission-dynamic models predicting the spread of COVID-19, we found the median estimated R_0_ to be 3.77, suggesting this novel virus is highly infectious. Estimated R_0_ of COVID-19 was higher than that of middle east respiratory syndrome coronavirus (MERS-CoV, <1) and severe acute respiratory syndrome (SARS, 2–4) (51, 52). This corresponds to the difference between the total number of infections seen in the current COVID-19 and 2003–2004 SARS outbreaks in China (>80,000 vs. ~5,327)^1^.

After a citywide lockdown began in Wuhan, the median estimated R_c_ dropped to 1.88, suggesting a large drop in infections coincided with the implementation of this outbreak control intervention. By limiting interactions and preventing travel, the lockdown effort has dramatically reduced contact rates between infected and non-infected persons. However, an R_c_>1 suggests that COVID-19 would continue to spread, which is at odds with recently published epidemiological reports, suggesting the outbreak in China is slowing down^1^. This discrepancy may suggest that other recently implemented public health measures beyond citywide lockdowns, including contact tracing, intensification of screening, quarantine of infected individuals, and mask utilisation, may also be contributing to the containment of COVID-19. Future models should attempt to capture the impact of these additional interventions on COVID-19 transmission.

We found median estimated incubation period, infectious period, and fatality were 5.90 days, 9.94 days, and 2.94%, respectively. If these estimations are accurate, a 14-day quarantine period would be long enough to assess for infection in an asymptomatic person exposed to COVID-19. It should be noted that the maximum incubation period reported for COVID-19 was 24 days, and additional research is needed to confirm these estimations. However, this estimated incubation period is similar to that of SARS and MERS (51, 52). Estimated CFR of COVID-19 (2.94%) was substantially lower than that of SARS (14–15%) and MERS (34.4%), suggesting COVID-19 may be a less virulent strain in the coronavirus family (20, 22, 51, 53). As of 9 May, the epidemic in China has almost come to end and local asymptomatic infected cases have been captured by the surveillance system, with the death toll come to 4,643 and fatality rate about 5.5%.

We found significant variation in estimated R_0_ and R_c_ in our review of the published literature. These variations may be due to the wide variety of modelling methods used and different assumptions used to build each model. Additionally, limited healthcare resources and immature diagnostic algorithms resulted in under-diagnosis and delayed treatment at the beginning of the outbreak in Wuhan. Consequently, models calibrated using only the official number of confirmed infections may be impacted by a systematic underreporting of infections, leading to a higher estimated R_0_ compared to models that adjusted for potential underreporting. Several studies included in this review concluded that underreported infections may have had an significant impact on estimated R_0_ (15, 21, 54), and five studies attempted to approximate the number of unreported cases (13, 18, 21, 22, 24).

Our mathematical model predicts peak time for COVID-19 will be in March 2020 and elimination is likely to be achieved by late April 2020 at the earliest, assuming the current intervention level is maintained. This estimate of peak time is close to the reality that there are few locally infected cases after 31 March, which indicates that the peak size of local transmission has been reached in March. Elimination of local transmission has been achieved in April as most of newly infected cases are imported from overseas. COVID-19 continues to spread worldwide^2^ (55), and the influx of overseas cases may introduce new transmission dynamics that are not possible to predict using current models. Studies have reported that the epidemic of some viruses (e.g., SARS-CoV) or bacteria (e.g., *Clostridioides difficile*) can be affected by geographical climatic factors such as temperature, humidity, and latitude (56–58), and COVID-19 infections may consequently be impacted by seasonality and latitude in unpredictable ways. However, we were not able to add additional results and analysis of temperature for COVID-19 due to lack of available data. In addition, the difference in social mixing pattern between rural and urban areas may lead to different transmission models. These data are essential to a thorough understanding of the spread of SARS-CoV-2 and formulating appropriate intervention strategies. Through comparing the two scenarios of our mathematical model, scenarios with an infectious incubation period resulted in much better goodness-of-fit. This to some extent support that incubation period is infectious. Finally, the potential impact of new treatment or vaccines for COVID-19 are not represented in the predictions of our models.

Our study has limitations. First, some studies included multiple models, and, as a result, models developed with certain underlying assumptions and validation methods were overrepresented in our results. Second, none of the included models considered age-related contact rates. Immunity to and fatality from COVID-19 likely differ across age cohorts. Without accounting for this key difference, results of all included models should be interpreted with caution. Third, our synthesis model did not take into account rates of underreported infections, additional quarantine efforts, mask usage, or changes in mass transportation, which may change our predictions. Without readily available data effects of these factors are hard to account for.

Findings from our systematic review and mathematical model suggest high infectiousness of COVID-19, and the lockdown of Wuhan significantly reduced R_0_. If current modelling is accurate, a 14-day quarantine is sufficient for asymptomatic persons exposed to the virus. The effect of age on infection and fatality should be incorporated into future models to more accurately predict transmission dynamics. Interventions besides citywide lockdowns, including mask utilisation and travel restrictions, should be further evaluated through modelling in order to better inform ongoing efforts to contact outbreaks inside and outside China.

## Data Availability Statement

All datasets generated for this study are included in the article/Supplementary Material.

## Author Contributions

HZo and YS conceived the study. Y-FL, QD, and YZho designed the protocol and conducted study selection and data extraction. Y-FL, QD, and YZho contributed to statistical analysis and interpretation of data. Y-FL, QD, YZho, TY, and PL drafted the manuscript. All authors critically revising the manuscript.

## Conflict of Interest

The authors declare that the research was conducted in the absence of any commercial or financial relationships that could be construed as a potential conflict of interest.

## Footnotes

^1^National Health Commission of the People's Republic of China. *Update on the Outbreak of Novel Coronavirus Pneumonia*. Available online at: www.nhc.gov.cn/ (accessed March 16, 2020).

^2^World Health Organization. *Coronavirus Disease (COVID-2019) Situation Reports*. Available online at: https://www.who.int/emergencies/diseases/novel-coronavirus-2019/situation-reports/ (accessed March 16, 2020).

^3^Tencent News. *Thirteen Cities in Hubei Implement “Lockdown” and Are Short of Medical Supplies*. Available online at: https://news.qq.com/a/20200124/013418.htm (accessed February 21, 2020).
