# Supplementary material for: Spread and Impact of COVID-19 in China: A Systematic Review and Synthesis of Predictions From Transmission-Dynamic Models
Source: Front Med (Lausanne). 2020 Jun 18;7:321. doi: 10.3389/fmed.2020.00321 (PMC7314927; doi:10.3389/fmed.2020.00321)
Supplement: Supplementary file 1 [file Data_Sheet_1.docx]

**Appendix**

| **Table S1. Quality assessment of included modeling studies** | | | | | | | | | | | | | | | | | |
| --- | --- | --- | --- | --- | --- | --- | --- | --- | --- | --- | --- | --- | --- | --- | --- | --- | --- |
| **Study** | **Aims and objectives** | **Setting and population** | **Intervention/comparators** | **Outcome measures** | **Model structure and time horizon** | **Modeling methods** | **Parameters, ranges and data sources** | **Assumptions explicit and justified** | **Quality of data and uncertainty and/or sensitivity analyses** | **Method of fitting** | **Model validation** | **Presentation of results and uncertainty** | **Interpretation and discussion of results** | **Funding source and conflicts of interest** | **Final Score (/28)** | **Rating** |  |
| A J Kucharski et al. | 2 | 2 | 1 | 2 | 2 | 2 | 2 | 1 | 0 | 2 | 0 | 2 | 2 | 2 | 22 | High |  |
| B Tang et al. | 2 | 2 | 2 | 2 | 2 | 2 | 2 | 2 | 2 | 2 | 0 | 2 | 2 | 2 | 26 | Very high |  |
| C You et al. | 2 | 2 | 1 | 2 | 2 | 2 | 2 | 1 | 0 | 2 | 0 | 2 | 2 | 2 | 22 | High |  |
| C Anastassopoulou et al. | 2 | 2 | 1 | 2 | 2 | 2 | 2 | 2 | 0 | 2 | 0 | 2 | 2 | 0 | 21 | High |  |
| H Xiong et al. | 2 | 2 | 1 | 2 | 2 | 2 | 2 | 1 | 0 | 2 | 0 | 2 | 2 | 2 | 22 | High |  |
| J M Read et al. | 2 | 2 | 1 | 0 | 2 | 2 | 2 | 0 | 0 | 2 | 0 | 2 | 2 | 0 | 17 | Medium |  |
| J T Wu et al. | 2 | 2 | 1 | 2 | 2 | 2 | 2 | 2 | 0 | 2 | 0 | 2 | 2 | 2 | 23 | Very high |  |
| J Riou et al. | 2 | 2 | 1 | 0 | 2 | 2 | 2 | 0 | 0 | 2 | 0 | 1 | 1 | 2 | 17 | Medium |  |
| K Mizumoto et al. | 2 | 2 | 1 | 0 | 2 | 2 | 2 | 0 | 0 | 1 | 0 | 2 | 2 | 2 | 18 | Medium |  |
| M Chinazzi et al. | 2 | 2 | 1 | 0 | 2 | 2 | 2 | 0 | 0 | 2 | 0 | 2 | 1 | 0 | 16 | Medium |  |
| M Shen et al. | 2 | 2 | 2 | 0 | 2 | 2 | 2 | 1 | 0 | 2 | 0 | 2 | 2 | 2 | 21 | High |  |
| S Zhao et al. | 2 | 2 | 1 | 1 | 2 | 2 | 2 | 0 | 0 | 2 | 0 | 2 | 2 | 2 | 20 | High |  |
| T Liu et al. | 2 | 2 | 1 | 1 | 2 | 2 | 2 | 1 | 0 | 2 | 0 | 2 | 2 | 0 | 19 | Medium |  |
| T Zhou et al. | 2 | 2 | 1 | 2 | 2 | 2 | 2 | 2 | 0 | 2 | 0 | 2 | 2 | 2 | 23 | Very high |  |
| X Li et al. | 2 | 2 | 1 | 2 | 2 | 2 | 2 | 1 | 0 | 2 | 0 | 2 | 2 | 2 | 22 | High |  |
| Z Cao et al. | 2 | 2 | 1 | 0 | 2 | 2 | 2 | 0 | 0 | 2 | 0 | 2 | 2 | 2 | 19 | Medium |  |
| W Wu et al. | 2 | 2 | 1 | 0 | 2 | 2 | 2 | 0 | 0 | 2 | 0 | 2 | 2 | 2 | 19 | Medium |  |
| S Zhao et al. | 2 | 2 | 1 | 1 | 2 | 2 | 2 | 0 | 0 | 2 | 0 | 2 | 2 | 2 | 20 | High |  |
| Q Zhao et al. | 2 | 2 | 2 | 1 | 2 | 2 | 2 | 0 | 0 | 2 | 0 | 2 | 2 | 2 | 21 | High |  |
| Y Yang et al. | 2 | 2 | 1 | 1 | 2 | 2 | 2 | 2 | 0 | 2 | 0 | 2 | 2 | 2 | 22 | High |  |
| S Sanche et al. | 2 | 2 | 1 | 0 | 2 | 2 | 2 | 0 | 0 | 1 | 0 | 2 | 2 | 2 | 18 | Medium |  |
| J Li et al. | 2 | 2 | 1 | 0 | 2 | 1 | 2 | 0 | 0 | 2 | 0 | 2 | 1 | 0 | 15 | Medium |  |
| S Jung et al. | 2 | 2 | 1 | 2 | 2 | 2 | 2 | 2 | 0 | 2 | 0 | 2 | 2 | 2 | 23 | Very high |  |
| Z Cao et al. | 2 | 2 | 1 | 0 | 2 | 2 | 2 | 0 | 0 | 2 | 0 | 2 | 2 | 2 | 19 | Medium |  |
| H Geng et al. | 2 | 2 | 2 | 1 | 2 | 2 | 2 | 0 | 0 | 2 | 0 | 2 | 2 | 0 | 19 | Medium |  |
| K Wan et al. | 2 | 2 | 1 | 1 | 2 | 2 | 2 | 1 | 0 | 2 | 0 | 2 | 1 | 2 | 20 | High |  |
| C Zhou et al. | 2 | 2 | 1 | 1 | 2 | 2 | 2 | 1 | 0 | 2 | 0 | 2 | 2 | 0 | 19 | Medium |  |

| **Table S2. Basic information of models targeting COVID-19** | | | | | |
| --- | --- | --- | --- | --- | --- |
| **Author** | **Model** | **Fitting target** | **Indicators** | **Region of interest** | **Value was considered before or after the closure (23 January 2020)** |
| W Ming | ODE based: SIR model | Reported infection | Intervention | China | After ^b^ |
| X Zhu | ODE based: SIR model | Reported infection | Total Infections; Peak time | China | After |
| Z Wang | ODE based: SIR model | Reported infection | Total Infections; Peak time | Hubei/China | After |
| W Wu | ODE based: SIR model | Reported infection | R_0_; Peak time | Other ^a^ | After |
| H Yuan | ODE based: SIR model | Reported infection | Intervention | China/Other | After |
| X Sun | ODE based: SIR model | Reported infection | R_0_ | China | After |
| A J Kucharski | ODE based: SEIR model | Reported infection | R_0_ | Wuhan | Before ^c^ (Scenario 1); After (Scenario 2) |
| B Tang | ODE based: SEIR model | Adjust reported infections | R_0_; Peak time and size;  Intervention | Wuhan | Before |
| H Wang | ODE based: SEIR model | Reported infection | Peak time and size; Elimination | China | After |
| J M. Read | ODE based: SEIR model | Reported infection | R_0_; Total Infections;  Infectious period | Wuhan | Before |
| J T Wu | ODE based: SEIR model | Adjust reported infections | R_0_; Peak time | Wuhan/Other | After |
| X Li | ODE based: SEIR model | Adjust reported infections | Peak time | Wuhan/Other | Before (Wuhan);  After (Other) |
| J Li | ODE based: SEIR model | Reported infection | R_0_ | China | After |
| L Peng | ODE based: SEIR model | Reported infection | Total Infections; Intervention | Wuhan/Hubei/China | After |
| H Geng | ODE based: SEIR model | Reported infection | R_0_ | China | After |
| L Ai | ODE based: SEIR model | Reported infection | Peak time and size | Hubei/China | After |
| K Wan | ODE based: SEIR model | Reported infection | R_0_; Infectious period; Incubation; Peak time, size; Elimination | Wuhan | After |
| K Wan | ODE based: SEIR model | Reported infection | R_0_; Peak time and size | Wuhan/Hubei | After |
| N Shao | ODE based: SEIR model | Reported infection | R_0_; Total infections | Wuhan/Hubei/China/Other | After |
| D Li | ODE based: Flow-SEIR model | Reported infection | Intervention | Hubei/China | After |
| Q Liu | ODE based: Flow-SEIR model | Stochastic simulations | Peak time and size;  Elimination; Intervention | Hubei/China | After |
| J Hellwell | Individual based: SEIR model | Reported infection | Intervention | China | After |
| M Shen | ODE based: SEIJR model | Reported infection | R_0_; Fatality; Incubation;  Total Infections;  Peak time and size | China | Before |
| M Shen | ODE based: SEIJR model | Reported infection | Incubation; Fatality;  Total Infections;  Peak time and size; Intervention | Hubei | After |
| T Zhou | ODE based: SEIJR model | Reported infection | R_0_ | China | Before |
| H Xiong | ODE based: EIR model | Reported infection | R_0_; Peal time and size;  Intervention | China | After |
| C Anastassopoulou | ODE based: SIRD model | Reported infection | R_0_; Infectious period; Fatality | Hubei | Before |
| Z Cao | ODE based: SEIRDC model | Reported infection | R_0_ | Wuhan | After |
| C Zhou | ODE based: SEIR and SEAIR model | Reported infection | R_0_ | Wuhan | After |
| S Zhao | EG model | Reported infection | R_0_ | Wuhan | Before |
| S W | EG model | Reported infection | Peak size | China | After |
| Z Cao | EG model | Reported infection | R_0_; Infectious period | China | After |
| S Jung | EG model | Reported infection | R_0_; Fatality | Wuhan | After |
| S Zhao | EG model | Reported and adjust reported infections | R_0_ | Wuhan | After |
| Q Zhao | EG model | Reported infection | R_0_; Infectious period | Wuhan | Before |
| T Liu | EG model | Adjust reported infections | R_0_; Incubation; Fatality;  Peak time | Wuhan/China/Other | After |
| S Sanche | EG model | Reported infection | R_0_ | Wuhan | After |
| J A. Backer | Probabilistic/likelihood-based model | Reported infection | Peak time and size | outside Wuhan | After |
| J Riou | Probabilistic/likelihood-based model | Stochastic simulations | R_0_ | Wuhan | Before |
| S A. Lauer | Probabilistic/likelihood-based model | Selection of cases | Incubation | China | NA |
| Y Yang | Probabilistic/likelihood-based model | Reported infection | R_0_; Incubation | Wuhan | After |
| C Leung | Probabilistic/likelihood-based model | Reported infection | Incubation | China | After |
| N M. Linton | Probabilistic/likelihood-based model | Selection of cases (outside of the epicenter of Hubei Province) | Incubation | China | After |
| K Mizumoto | Probabilistic/likelihood-based model | Reported infection | R_0_; Total Infections | Wuhan | Before (laboratory confirmed cases);  After (epidemiological data) |
| S Ai | Probabilistic/likelihood-based model | Reported infection | Intervention | Wuhan | After |
| M Batista | Probabilistic/likelihood-based model | Reported infection | R_0_; Total Infections | China | After |
| X Fu | Probabilistic/likelihood-based model | Reported infection | Total Infections | Wuhan/Hubei/China/Other | After |
| M Chinazzi | Probabilistic/likelihood-based model | Reported infection | R_0_ | Wuhan | Before |
| G Zhou | Probabilistic/likelihood-based model | Reported infection | R_0_; Total Infections | China | After |
| J Li | EG model; Probabilistic/likelihood-based model; ODE based: SEIR model | Reported infection | R_0_ | Wuhan | After |
| C You | Probabilistic/likelihood-based model;  EG model; ODE based: SIR model | Reported infection | R_0_; Infectious period | China/Hubei/Other | After |
| T Zeng | ODE based model | Reported infection | R_0_; Total Infections; Peak time | China | After |
| Footnote: ^a^ Other regions other than Hubei in China; ^b^ infection data after 23 January 2020 were used; ^c^ only infection data before 23 January 2020 were used.  Abbreviation: ODE, Ordinal Differential Equation; SIR, Susceptible-Infected-Recovered; SEIR, Susceptible-Exposed-Infectious-Recovered; SEIJR, Susceptible-Exposed-Infectious-Isolated-Recovered; EIR, Exposed-Infectious-Recovered; SIRD, Susceptible-Infected-Recovered-Dead; SEIRDC, Susceptible-Exposed-Infectious-Recovered-Dead-Auxiliary variable; SEAIR, Susceptible-Exposed-Asymptomatic-Infectious-Recovered; EG, Exponential Growth; R_0_, the reproduction number. | | | | | |

**Introduction of models in this systematic review**

Models were classified into three categories: (1) ordinary differential equation (ODE) systems, (2) exponential growing (EG) models, and (3) Probabilistic/likelihood-based (PB) models. ODE, EG, and PB models accounted for 57% (32/56), 18% (10/56), and 25% (14/56) of articles, respectively. PB based models, including machine learning techniques and stochastic simulations, were used but not mainly in predicting future trends. (1-7) Among dynamic transmission models, the SEIR structure was used most frequently,(5, 8-11) with adjustments to this structure based on individual research questions.(12-16) EG and PB models were fit to empirical data and computed parameters of interest directly; however, these models also assumed the epidemic would follow a susceptible-infectious-recovery (SIR) or SEIR dynamic.

Most data used for model fitting and calibration came from three sources: (1) the number of confirmed cases within Wuhan/Hubei reported by the National Health Commission of China, (17) (2) the adjusted number of cases in Wuhan/Hubei, including cases identified in other provinces but found to have originated in Wuhan/Hubei,(2, 10) and (3) the adjusted number of cases in Wuhan/Hubei after correcting for differential reporting rates across Wuhan/Hubei. (18, 19) Data included cases reported between 1 December 2019 and 10 February 2020. Several studies used other sources of data (10, 20, 21), including global airline traffic flow to and from Wuhan, domestic passenger volumes to and from Wuhan during the Chinese New Year travel season, and real-time travel data from the Baidu® Migration server.

| **Table S3. Basic reproduction number (R_0_) extracted from models** | | | | | |
| --- | --- | --- | --- | --- | --- |
| **First author** | **Model** | **Mean/Median** | **CI/Range** | **Region of interest** | **R was considered before or after the lockdown (23 January 2020)** |
| W Wu | ODE based: SIR model | 2.23 | (1.54, 2.92) | Other * | R_0_ |
| W Wu | ODE based: SIR model | 1.91 | (1.48, 2.34) | Other | R_c_ |
| C You | ODE based: SIR model | 2.30 | (1.10, 3.90) | China | R_0_ |
| C You | ODE based: SIR model | 5.00 | (3.30, 7.00) | Hubei | R_0_ |
| C You | ODE based: SIR model | 5.40 | (4.60, 6.20) | Other | R_0_ |
| C You | ODE based: SIR model | 2.10 | (1.00, 3.30) | China | R_c_ |
| C You | ODE based: SIR model | 1.00 | (0.40, 1.70) | Hubei | R_c_ |
| C You | ODE based: SIR model | 1.50 | (1.10, 2.00) | Other | R_c_ |
| H Sun | ODE based: SIR model | 7.67 | (5.21, 10.13) | Hubei | R_0_ |
| H Sun | ODE based: SIR model | 6.24 | (4.67, 7.81) | China | R_0_ |
| H Sun | ODE based: SIR model | 1.01 | (0.58, 1.44) | Hubei | R_c_ |
| H Sun | ODE based: SIR model | 0.77 | (0.34, 1.20) | China | R_c_ |
| A J Kucharski | ODE based: SIRD mode | 2.35 | (1.15, 4.77) | Wuhan | R_0_ |
| A J Kucharski | ODE based: SIRD mode | 1.05 | (0.41, 2.39) | Wuhan | R_c_ |
| B Tang | ODE based: SEIR model | 6.47 | (5.71, 7.23) | Wuhan | R_0_ |
| J M. Read | ODE based: SEIR model | 3.11 | (2.39, 4.13) | Wuhan | R_0_ |
| J T Wu | ODE based: SEIR model (baseline) | 2.68 | (2.47, 2.86) | Wuhan | R_0_ |
| J T Wu | ODE based: SEIR model (Model 1) | 2.53 | (2.32, 2.71) | Wuhan | R_0_ |
| J T Wu | ODE based: SEIR model (Model 2) | 2.42 | (2.22, 2.60) | Wuhan | R_0_ |
| X Li | ODE based: SEIR model | 5.00 | NA | Wuhan | R_0_ |
| X Li | ODE based: SEIR model | 1.40 | (1.19, 1.61) | Beijing | R_c_ |
| K Wan | ODE based: SEIR model | 3.00 | (2.80, 3.10) | China | R_c_ |
| J Li | ODE based: SEIR model | NA | (8.00, 14.00) | 84 cities in China | R_0_ |
| Z Cao | ODE based: SEIR model | 3.24 | NA | Wuhan | R_0_ |
| H Geng | ODE based: SEIR model | NA | (2.38, 2.72) | China mainland | R_c_ |
| K Wan | ODE based: SEIR model | 1.44 | (1.40, 1.47) | Wuhan | R_c_ |
| C Zhou | ODE based: SEIR model | 2.12 | (2.04, 2.18) | Wuhan | R_c_ |
| H Xiong | ODE based: SEIR model | 2.70 | NA | China | R_0_ |
| C You | Probabilistic/likelihood-based model | 3.02 | (2.02, 4.42) | China | R_0_ |
| C You | Probabilistic/likelihood-based model | 3.33 | (2.12, 5.06) | Hubei | R_0_ |
| C You | Probabilistic/likelihood-based model | 2.68 | (1.75, 3.94) | Other | R_0_ |
| C You | Probabilistic/likelihood-based model | 2.28 | (1.73, 2.99) | China | R_c_ |
| C You | Probabilistic/likelihood-based model | 2.50 | (1.91, 3.32) | Hubei | R_c_ |
| C You | Probabilistic/likelihood-based model | 1.84 | (1.47, 2.33) | Other | R_c_ |
| C You | EG model | 3.74 | (2.50, 6.01) | China | R_0_ |
| C You | EG model | 3.76 | (2.05, 7.44) | Hubei | R_0_ |
| C You | EG model | 3.69 | (2.28, 6.47) | Other | R_0_ |
| C You | EG model | 1.65 | (1.46, 1.91) | China | R_c_ |
| C You | EG model | 1.94 | (1.64, 2.37) | Hubei | R_c_ |
| C You | EG model | 1.62 | (0.86, 2.99) | Other | R_c_ |
| S Zhao | EG model | 2.56 | (2.49, 2.63) | Wuhan | R_0_ |
| T Liu | EG model | 4.50 | (4.40, 4.60) | China | R_0_ |
| T Liu | EG model | 4.40 | (4.30, 4.60) | Wuhan | R_0_ |
| T Liu | EG model | 0.60 | (0.40, 0.70) | Other | R_c_ |
| C Anastassopoulou | ODE based: SIRD mode (Scenario 1 on 16 Jan) | 4.80 | (3.36, 6.67) | Hubei | R_0_ |
| C Anastassopoulou | ODE based: SIRD mode (Scenario 1 on 17 Jan) | 4.60 | (3.56, 5.65) | Hubei | R_0_ |
| C Anastassopoulou | ODE based: SIRD mode (Scenario 1 on 18 Jan) | 5.14 | (4.25, 6.03) | Hubei | R_0_ |
| C Anastassopoulou | ODE based: SIRD mode (Scenario 1 on 19 Jan) | 6.09 | (5.02, 7.16) | Hubei | R_0_ |
| C Anastassopoulou | ODE based: SIRD mode (Scenario 1 on 20 Jan) | 7.09 | (5.84, 8.35) | Hubei | R_0_ |
| C Anastassopoulou | ODE based: SIRD mode (Scenario 2 on 16 Jan) | 4.15 | (2.92, 5.38) | Hubei | R_0_ |
| C Anastassopoulou | ODE based: SIRD mode (Scenario 2 on 17 Jan) | 3.98 | (3.11, 4.85) | Hubei | R_0_ |
| C Anastassopoulou | ODE based: SIRD mode (Scenario 2 on 18 Jan) | 4.39 | (3.67, 5.11) | Hubei | R_0_ |
| C Anastassopoulou | ODE based: SIRD mode (Scenario 2 on 19 Jan) | 5.15 | (4.30, 6.01) | Hubei | R_0_ |
| C Anastassopoulou | ODE based: SIRD mode (Scenario 2 on 20 Jan) | 6.01 | (4.93, 7.08) | Hubei | R_0_ |
| C Zhou | ODE based: SEAIR model | 2.05 | NA | Wuhan | R_c_ |
| H Xiong | ODE based: EIR model | 2.70 | NA | China | R_c_ |
| M Shen | ODE based: SEIJR model | 4.71 | (4.50, 4.92) | China | R_0_ |
| T Zhou | ODE based: SEIJR model | NA | (2.80, 3.90) | China | R_c_ |
| J Riou | Probabilistic/likelihood-based model | 2.20 | (1.40, 3.80) | Wuhan | R_0_ |
| K Mizumoto | Probabilistic/likelihood-based model | 7.05 | (6.11, 8.18) | Wuhan | R_0_ |
| K Mizumoto | Probabilistic/likelihood-based model | 3.24 | (3.16, 3.32) | Wuhan | R_c_ |
| M Chinazzi | Probabilistic/likelihood-based model | 2.40 | (2.20, 2.60) | Wuhan | R_0_ |
| Y Yang | Probabilistic/likelihood-based model | 3.77 | (3.51, 4.05) | Wuhan | R_c_ |
| T Liu | Probabilistic/likelihood-based model | 4.50 | (4.30, 4.60) | China | R_0_ |
| T Liu | Probabilistic/likelihood-based model | 4.40 | (4.30, 4.60) | Wuhan | R_0_ |
| T Liu | Probabilistic/likelihood-based model | 0.60 | (0.40, 0.70) | Other | R_c_ |
| T Zhou | Probabilistic/likelihood-based model | NA | (2.80, 3.90) | China | R_0_ |
| Y Yang | Probabilistic/likelihood-based model | 3.77 | (3.51, 4.05) | Wuhan | R_0_ |
| N Shao | Probabilistic/likelihood-based model | 3.12 | NA | Wuhan | R_0_ |
| N Shao | Probabilistic/likelihood-based model | 3.02 | NA | Hubei | R_0_ |
| N Shao | Probabilistic/likelihood-based model | 3.04 | NA | China | R_0_ |
| Z Cao | EG model | 4.08 | (3.37, 4.77) | China | R_c_ |
| S Zhao | EG model | 2.24 | (1.96, 2.55) | Wuhan | R_0_ |
| S Zhao | EG model | 3.58 | (2.89, 4.39) | Wuhan | R_0_ |
| S Zhao | EG model | 5.71 | (4.24, 7.54) | Wuhan | R_0_ |
| Q Zhao | EG model | 5.70 | (3.40, 9.20) | Wuhan | R_0_ |
| Q Zhao | EG model | 5.10 | (3.40, 7.50) | Wuhan | R_0_ |
| S Sanch | EG model | NA | (4.70, 6.60) | Wuhan | R_0_ |
| S Jung | EG model | 2.10 | (2.00, 2.20) | Wuhan | R_0_ |
| S Jung | EG model | 3.20 | (2.70, 3.70) | Wuhan | R_0_ |
| J Li | ODE based: SEIR model | 4.38 | (3.63, 5.13) | Wuhan | R_0_ |
| J Li | ODE based: SEIR model | 3.41 | (3.16, 3.65) | Wuhan | R_c_ |
| Footnote: * Other regions other than Hubei in China.  Abbreviation: ODE, Ordinal Differential Equation; SIR, Susceptible-Infected-Recovered; SIRD, Susceptible-Infected-Recovered-Dead; SEIR, Susceptible-Exposed-Infectious-Recovered; EG, Exponential Growth; SEAIR, Susceptible-Exposed-Asymptomatic-Infectious-Removed; EIR, Exposed-Infectious-Recovered; SEIJR, Susceptible-Exposed-Infectious-Isolated-Recovered; R_0_, Basic reproduction number; R_c_, Controlled reproduction number. | | | | | |

| **Table S4. Indicators extracted from models (Incubation, Infectious period, Total infections and Fatality)** | | | | |  |
| --- | --- | --- | --- | --- | --- |
| **First author** | **Model** | **Mean/Median** | **CI/Range** | **Region of interest** |  |
| **Incubation** |  |  |  |  |  |
| K Wan | ODE based: SEIR model | 3 | (2.80, 3.10) | China |  |
| M Shen | ODE based: SEIJR model (isolation) | 5.03 | (4.93, 5 .12) | China |  |
| M Shen | ODE based: SEIJR model (lockdown) | 5.2 | (5.20, 5.20) | China |  |
| T Liu | EG model | 4.8 | (1.00, 14.00) | Other * |  |
| J A. Backer | Probabilistic/likelihood-based model | 6.4 | (5.50, 7.50) | Wuhan |  |
| J A. Backer | Probabilistic/likelihood-based model | 6.1 | (5.30, 7.30) | Wuhan |  |
| J A. Backer | Probabilistic/likelihood-based model | 6.1 | (5.20, 7.40) | Wuhan |  |
| S A. Lauer | Probabilistic/likelihood-based model | 7.92 | (3.97, 24.98) | China |  |
| S A. Lauer | Probabilistic/likelihood-based model | 3.11 | (2.20, 6.08) | China |  |
| S A. Lauer | Probabilistic/likelihood-based model | 14 | (5.00, 21.00) | China |  |
| Y Yang | Probabilistic/likelihood-based model | 4.75 | (3.00, 7.20) | China |  |
| C Leung | Probabilistic/likelihood-based model | 1.7 | (0.00, 3.44) | China |  |
| C Leung | Probabilistic/likelihood-based model | 1.8 | (0.17, 3.43) | China |  |
| C Leung | Probabilistic/likelihood-based model | 1.8 | (0.17, 3.43) | China |  |
| C Leung | Probabilistic/likelihood-based model | 17.1 | (5.93, 28.27) | China |  |
| C Leung | Probabilistic/likelihood-based model | 6.9 | (0.00, 14.87) | China |  |
| C Leung | Probabilistic/likelihood-based model | 6.9 | (0.00, 15.33) | China |  |
| N M. Linton | Probabilistic/likelihood-based model | 5 | (4.20, 6.00) | China |  |
| N M. Linton | Probabilistic/likelihood-based model | 5.4 | (4.30, 6.60) | China |  |
| N M. Linton | Probabilistic/likelihood-based model | NA | (4.30, 6.60) | China |  |
| N M. Linton | Probabilistic/likelihood-based model | NA | (5.00, 6.30) | China |  |
| N M. Linton | Probabilistic/likelihood-based model | 5.8 | (5.20, 6.50) | China |  |
| N M. Linton | Probabilistic/likelihood-based model | 6 | (5.30, 6.70) | China |  |
| **Infectious period** |  |  |  |  |  |
| C Anastassopoulou | ODE based: SIRD mode (Scenario 1) | 20 | (18.00, 22.00) | China |  |
| C Anastassopoulou | ODE based: SIRD mode (Scenario 2) | 12 | (11.00, 13.00) | China |  |
| J M. Read | ODE based: SEIR model | 1.6 | (0.35, 3.23) | Wuhan |  |
| K Wan | ODE based: SEIR model | 14 | NA | Wuhan |  |
| Z Cao | EG model | NA | (0.00, 2.30) | China |  |
| C You | Probabilistic/likelihood-based model; EG model; ODE based: SIR model | 10.91 | (3.17, 18.65) | China |  |
| **Total infections** |  |  |  |  |  |
| X Zhu | ODE based: SIR model (Scenario 2) | 14137 | NA | China |  |
| X Zhu | ODE based: SIR model (Scenario 3) | 411082 | NA | China |  |
| Z Wang | ODE based: SIR model | 75000 | NA | China |  |
| Z Wang | ODE based: SIR model | 37000 | NA | Hubei |  |
| J M. Read | ODE based: SEIR model | 21022 | (11090, 33490) | Wuhan |  |
| L Peng | ODE based: SEIR model | NA | (12900, 160000) | Other |  |
| L Peng | ODE based: SEIR model | NA | (20000, 260000) | Hubei |  |
| L Peng | ODE based: SEIR model | Over 55000 | NA | Wuhan |  |
| M Shen | ODE based: SEIJR model (Isolation) | 8042 | (4199, 11884) | China |  |
| M Shen | ODE based: SEIJR model (lockdown) | 128960 | (39362, 218560) | Hubei |  |
| M Shen | ODE based: SEIJR model (lockdown) | 348750 | (84140, 613360) | China |  |
| T Zeng | ODE based model | 46000 | NA | China |  |
| K Mizumoto | Probabilistic/likelihood-based model | 983006 | (759475, 1296258) | Wuhan |  |
| M Batista | Probabilistic/likelihood-based model | 1200000 | NA | China |  |
| X Fu | Probabilistic/likelihood-based model | 79467 | (71348, 96569) | China |  |
| X Fu | Probabilistic/likelihood-based model | 64261 | (57993, 76056) | Hubei |  |
| X Fu | Probabilistic/likelihood-based model | 46607 | (41245, 58129) | Wuhan |  |
| X Fu | Probabilistic/likelihood-based model | 13906 | (12727, 15901) | Other |  |
| N Shao | Probabilistic/likelihood-based model | 48060 | NA | Wuhan |  |
| N Shao | Probabilistic/likelihood-based model | 78480 | NA | China |  |
| **Fatality** |  |  |  |  |  |
| C Anastassopoulou | ODE based: SIRD mode (Scenario 1) | 2.94 | (2.89, 3.00) | China |  |
| C Anastassopoulou | ODE based: SIRD mode (Scenario 2) | 0.58 | (0.57, 0.59) | China |  |
| M Shen | ODE based: SEIJR model (Isolation) | 11.02 | (9.26, 12.78) | China |  |
| M Shen | ODE based: SEIJR model (lockdown) | 3.44 | (3.19, 3.70) | China |  |
| T Liu | EG model | 2.8 | NA | Hubei |  |
| T Liu | EG model | 0.24 | NA | Other |  |
| S Jung | EG model | 8.4 | (5.30, 12.30) | Wuhan |  |
| K Mizumoto | Probabilistic/likelihood-based model | 4.5 | (4.02, 5.31) | Wuhan |  |
| Footnote: * Other regions other than Hubei in China.  Abbreviation: ODE, Ordinal Differential Equation; SEIR, Susceptible-Exposed-Infectious-Recovered; SEIJR, Susceptible-Exposed-Infectious-Isolated-Recovered; EG, Exponential Growth; SIRD, Susceptible-Infected-Recovered-Dead; SIR, Susceptible-Infected-Recovered. | | | | | |

| **Table S5. Intervention Summary** | | | |
| --- | --- | --- | --- |
| **First author** | **Model** | **Method** | **Result** |
| W Ming | ODE ^a^ based: SIR ^b^ model | Efficacy of diagnosis (rate) | If 70% efficacy rate could be achieved, the forecasting number of cases would drop dramatically to 11,056 as of 10 February compared to 115,355 without public health interventions. |
| H Yuan | ODE based: SIR model | Lockdown | To make an extra 30 days gain, under the low or high reproduction number (1.4 or 2.92), the control measures have to reduce 87% or 95% of the secondary infections generated by the imported cases. |
| J Hellwell | Individual based: SEIR ^c^ model | Isolation | If 80% of contacts to be traced and isolated with a reproduction number of 2.5, 90% of the outbreaks can be controlled. |
| B Tang | ODE based: SEIR model | Quarantine and Isolation (rate) | Increasing quarantine rate by 5, 10, 15, or 20 folds will bring forward the peak by 4.2, 6.5, 7.9 or 9 days, and lead to a reduction of the peak value in terms of the number of infected individuals by 76.9%, 87.9%, 91.5%, or 93.3%. |
| H Geng | ODE based: SEIR model | Lockdown | The infection curve slowed down smoothly, and the peak value of latent and infected population decreased by 45.71% and 29.90%, respectively, after travel restriction. |
| Q Liu | ODE based: Flow-SEIR model | Self-protection and Isolation | If all people were in a completely ideal state of self-protection and isolation, the peak will decrease by 89.68% on average. If not, the peak will increase by 20.4%. |
| D Li | ODE based: Flow-SEIR model | Lockdown | If the intervention was 1 day of delay, about 1,800 people will finally be at risk. However, provincial level traffic blockage can only alleviate 21.06%–22.38% of the peak number of the infections. |
| H Xiong | ODE based: EIR ^d^ model | Lockdown | If the starting date of intervention is delayed for 1, 3, or 7 days than the date 23 January, the peak infected population will increase about 6,351, 21,621, or 65,929 individuals. |
| M Shen | ODE based: SEIJR ^e^ model | Lockdown | This suggests that the current lockdown strategy may eventually prevent 219,790 (44,773–394,800) 2019-nCoV infections and 6,695 (1,741–11,650) deaths—that is, 62.53% (59.98–65.08%) of infections and 59.91% (56.87–62.95%) deaths, compared with no intervention. Further, if the lockdown has been delayed for 7 or 14 days, the number of confirmed cases at peak will increase to 18,095 (8,618–28,274) and 25,194 (11,634–39,667). |
| S Ai | Probabilistic/likelihood-based model | Lockdown | If the intervention was delayed by 1 or 2 days, there would be 722 or 1,462 extra 2019-nCoV cases respectively. |
| Abbreviation: ODE, Ordinal Differential Equation; SIR, Susceptible-Infected-Recovered; SEIR, Susceptible-Exposed-Infectious-Recovered; EIR, Exposed-Infectious-Recovered; SEIJR, Susceptible-Exposed-Infectious-Isolated-Recovered. | | | |

**Model formulation**

We modelled 2019-nCoV transmission to predict the future trend and expected peak date in Wuhan. The population was divided into four compartments: susceptible(S), asymptomatic individuals during the incubation period (E), infectious individuals with symptoms (I) and recovered individuals (R) - (SEIR) structure (see below figure).


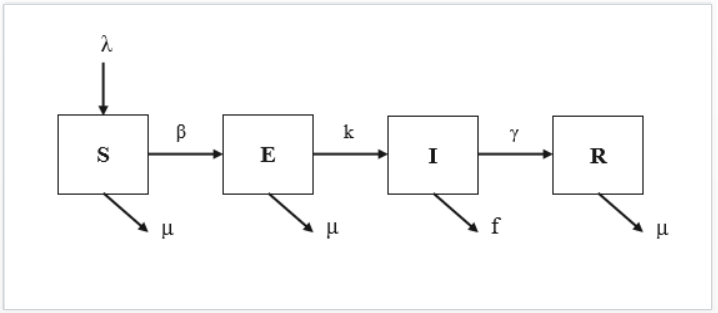


The susceptible individuals became infected by being in contact with the infectious individuals and entered the latent compartment at the transmission rate per day, 𝛽. Individuals in the incubation period progressed to the infectious compartment at a rate of k. Infectious individuals recovered or died at the rate of 𝛾 or f. The natural birth and death rates are λ and 𝜇.

The transmission model, with two assumptions, can be represented as a linked system of ordinary differential equations to track the rates of change in the number of people in each health state:

Assumption 1: Individuals in incubation period are infectious.

S: $dS=\lambda*N-\beta*S*(I+E)/N-\mu*S$

E: $dE=\beta*S*(I+E)/N-k*E-\mu*E$

I: $dI=k*E-\gamma*I-f*I$

R: $dR=\gamma*I-\mu*R$

Assumption 2: Individuals in incubation period are NOT infectious.

S: $dS=\lambda*N-\beta*S*I/N-\mu*S$

E: $dE=\beta*S*I/N-k*E-\mu*E$

I: $dI=k*E-\gamma*I-f*I$

R: $dR=\gamma*I-\mu*R$

Where N is the total population (N = S + E + I + R).

No interventions were considered in this model, but we assumed the current intervention(s) will effectively control R_0_ and make it an exponential decline over time ($R_{t}=R_{t-1}*\alpha^{t}$ where t is the days after the initial date and α is the exponential decline rate). The dynamic rate at which susceptible individuals, 𝛽_t_, become infected was determined and calculated via

$$\beta_{t}\approx R_{t}\times\gamma= \frac{R_{t}}{Infecticous period}$$

For each four parameters, including reproduction number, incubation period, infectious period and fatality, four values [1^st^ quantile (Q1), median, mean, and 3^rd^ quantile (Q3)] were extracted and inputted our model. In the calibration, the top 20 of 256 best-fit simulations, according to the least mean square error, were used to obtain the best estimates of the epidemic trends. The peak times and eliminations (total infections < 100) time based on normal (median), optimistic and pessimistic scenarios (sensitivity analyses) were our interesting.

All analyses were conducted with R software version 3.6.2 (R Foundation for Statistical Computing), and confirmed reported cases between 12–21 February 2020 were download from the China National Health Commission.

| **Table S6. Model parameters, estimated values and forecasts for China under two assumptions** | | | | |
| --- | --- | --- | --- | --- |
| **Parameter** | **Assumption 1** | | **Assumption 2** | |
|  | **Estimated values** | **SD/Range** | **Estimated values** | **SD/Range** |
| **R_0_** | 2.15 | 0.15 | 2.14 | 0.16 |
| **Incubation** | 5.19 | 0.53 | 5.17 | 0.50 |
| **Infectious period** | 11.87 | 1.35 | 12.00 | 1.51 |
| **Fatality** | 2.68% | 0.67% | 2.43% | 0.39% |
| **Peak time** | 17 March | 12-22 March | 2 March | 13 February - 5 Mar |
| **Elimination** | 7 May | 25 April – 21 May | 17 May | 8-27 May |
| **Mean square error (20 best-fit)** | 17,944 | 1,140 | 27,750 | 1,754 |
| Footnote: Assumption 1, individuals in incubation period are infectious; assumption 2, Individuals in incubation period are not infectious. Abbreviation: R_0_, the reproduction number; SD, standard deviation. | | | | |

**Reference**

1. Riou J, Althaus CL. Pattern of early human-to-human transmission of Wuhan 2019 novel coronavirus (2019-nCoV), December 2019 to January 2020. *Euro surveillance : bulletin Europeen sur les maladies transmissibles = European communicable disease bulletin* (2020) 25(4). doi: 10.2807/1560-7917.ES.2020.25.4.2000058.

2. Chinazzi M, Davis JT, Ajelli M, Gioannini C, Litvinova M, Merler S, et al. The effect of travel restrictions on the spread of the 2019 novel coronavirus (2019-nCoV) outbreak. *medRxiv* (2020):2020.02.09.20021261. doi: 10.1101/2020.02.09.20021261.

3. Yang X, Xu T, Jia P, Xia H, Guo L, Ye K. Transportation, Germs, Culture: A Dynamic Graph Model of 2019-nCoV Spread. (2020).

4. Zhu X, Zhang A, Xu S, Jia P, Tan X, Tian J, et al. Spatially Explicit Modeling of 2019-nCoV Epidemic Trend based on Mobile Phone Data in Mainland China. *medRxiv* (2020):2020.02.09.20021360. doi: 10.1101/2020.02.09.20021360.

5. Li X, Zhao X, Sun Y. The lockdown of Hubei Province causing different transmission dynamics of the novel coronavirus (2019-nCoV) in Wuhan and Beijing. *medRxiv* (2020):2020.02.09.20021477. doi: 10.1101/2020.02.09.20021477.

6. Chen Y, Cheng J, Jiang Y, Liu K. A Time Delay Dynamic System with External Source for the Local Outbreak of 2019-nCoV. *arXiv e-prints* [Internet]. (2020 February 01, 2020). Available from: <https://ui.adsabs.harvard.edu/abs/2020arXiv200202590C>.

7. Cao Z, Zhang Q, Lu X, Pfeiffer D, Jia Z, Song H, et al. Estimating the effective reproduction number of the 2019-nCoV in China. *medRxiv* (2020):2020.01.27.20018952. doi: 10.1101/2020.01.27.20018952.

8. Tang B, Wang X, Li Q, Bragazzi NL, Tang S, Xiao Y, et al. Estimation of the Transmission Risk of the 2019-nCoV and Its Implication for Public Health Interventions. *J Clin Med* (2020) 9(2). Epub 2020/02/13. doi: 10.3390/jcm9020462. PubMed PMID: 32046137.

9. Read JM, Bridgen JRE, Cummings DAT, Ho A, Jewell CP. Novel coronavirus 2019-nCoV: early estimation of epidemiological parameters and epidemic predictions. *medRxiv* (2020):2020.01.23.20018549. doi: 10.1101/2020.01.23.20018549.

10. Wu JT, Leung K, Leung GM. Nowcasting and forecasting the potential domestic and international spread of the 2019-nCoV outbreak originating in Wuhan, China: a modelling study. *The Lancet* (2020). doi: 10.1016/S0140-6736(20)30260-9.

11. Li J. A Robust Stochastic Method of Estimating the Transmission Potential of 2019-nCoV. *arXiv e-prints* [Internet]. (2020 February 01, 2020). Available from: <https://ui.adsabs.harvard.edu/abs/2020arXiv200203828L>.

12. Shen M, Peng Z, Guo Y, Xiao Y, Zhang L. Lockdown may partially halt the spread of 2019 novel coronavirus in Hubei province, China. *medRxiv* (2020):2020.02.11.20022236. doi: 10.1101/2020.02.11.20022236.

13. Shen M, Peng Z, Xiao Y, Zhang L. Modelling the epidemic trend of the 2019 novel coronavirus outbreak in China. *bioRxiv* (2020):2020.01.23.916726. doi: 10.1101/2020.01.23.916726.

14. Zhou T, Liu Q, Yang Z, Liao J, Yang K, Bai W, et al. Preliminary prediction of the basic reproduction number of the Wuhan novel coronavirus 2019-nCoV. *J Evid Based Med* (2020). Epub 2020/02/13. doi: 10.1111/jebm.12376. PubMed PMID: 32048815.

15. Cao Z, Zhang Q, Lu X, Pfeiffer D, Wang L, Song H, et al. Incorporating Human Movement Data to Improve Epidemiological Estimates for 2019-nCoV. *medRxiv* (2020):2020.02.07.20021071. doi: 10.1101/2020.02.07.20021071.

16. Zhou C. Evaluating new evidence in the early dynamics of the novel coronavirus COVID-19 outbreak in Wuhan, China with real time domestic traffic and potential asymptomatic transmissions. *medRxiv* (2020):2020.02.15.20023440. doi: 10.1101/2020.02.15.20023440.

17. National Health Commission of the People's Republic of China. Update on the outbreak of novel coronavirus pneumonia [cited 2020 March 16]. Available from: [www.nhc.gov.cn/](file:///C:\Users\giorgia.aprile\Downloads\www.nhc.gov.cn\).

18. Anastassopoulou C, Russo L, Tsakris A, Siettos C. Data-Based Analysis, Modelling and Forecasting of the novel Coronavirus (2019-nCoV) outbreak. *medRxiv* (2020):2020.02.11.20022186. doi: 10.1101/2020.02.11.20022186.

19. Zhao S, Lin Q, Ran J, Musa SS, Yang G, Wang W, et al. Preliminary estimation of the basic reproduction number of novel coronavirus (2019-nCoV) in China, from 2019 to 2020: A data-driven analysis in the early phase of the outbreak. *bioRxiv* (2020):2020.01.23.916395. doi: 10.1101/2020.01.23.916395.

20. Lai S, Bogoch I, Ruktanonchai N, Watts A, Li Y, Yu J, et al. Assessing spread risk of Wuhan novel coronavirus within and beyond China, January-April 2020: a travel network-based modelling study. *medRxiv* (2020):2020.02.04.20020479. doi: 10.1101/2020.02.04.20020479.

21. Ai S, Zhu G, Tian F, Li H, Gao Y, Wu Y, et al. Population movement, city closure and spatial transmission of the 2019-nCoV infection in China. *medRxiv* (2020):2020.02.04.20020339. doi: 10.1101/2020.02.04.20020339.
